# Relationships between self perceptions and physical activity behaviour, fear of falling, and physical function among older adults

**DOI:** 10.1186/s11556-017-0185-3

**Published:** 2017-09-20

**Authors:** Myrla Sales, Pazit Levinger, Remco Polman

**Affiliations:** 1 Institute of Sport, Exercise & Active Living (ISEAL), College of Sport and Exercise Science, Victoria University, Melbourne, Victoria Australia; 2 Associate Professor and Group Discipline Leader Exercise Science and Clinical Rehabilitation, Institute of Sport, Exercise & Active Living (ISEAL), College of Sport and Exercise Science, Victoria University, Melbourne, Victoria Australia; 3 Professor and Head of School Exercise & Nutrition Sciences, Queensland University of Technology, Kelvin Grove Campus, Brisbane, Australia

**Keywords:** Self-perceptions, Fear of falling, Muscle strength, Physical functioning

## Abstract

**Background:**

There has been a lack of research examining the relationship among self-perceptions, behaviour, cognitions and functioning in older adults. This study, therefore, examined the relationship between global and physical self-perceptions, physical activity behaviour, and fear of falling taking into considerations objective measures of physical functioning in community dwelling older adults.

**Methods/design:**

Sixty-six participants between 60 and 90 years old (71.9 ± 6.6 years; 47 females; 19 males) completed questionnaires assessing physical and global self-description (PSDQ), planned and incidental physical activity behaviour (IPEQ), and falls efficacy (Short FES-I) as well as tests measuring physical functioning. Backwards multiple linear regression modelling was used to assess possible relationships among variables.

**Results:**

Findings showed that physical self-perceptions (activity, coordination, endurance, flexibility) were associated with self-reported planned and incidental PA whereas sit-to-stand was the only objectively measured physical functioning variable associated with planned PA. Similarly, more falls, global self-esteem, general physical and domain specific physical self-perceptions (flexibility and strength) as well as knee strength were associated with fear of falling. There were also associations between some of the objectively measured physical functioning variables and self-perceptions of the physical self, providing some predictive validity for the PDSQ.

**Conclusions:**

The findings of this study come to corroborate that the belief system of older adults ideally need to be taken into consideration when designing interventions that aim to enhance PA behaviour or reduce fear of falling. Coupling that with goal-setting, life coaching and behaviour change strategies would also be beneficial to address engagement and adherence to such interventions.

**Trial registration:**

This trial was retrospectively registered with the Australian New Zealand Clinical Trials Registry - Registry No. ACTRN12614000700639 on the Jul 03rd 2014.

## Background

Perception of the physical self which includes appearance, function and ability to perform physical activities, may influence physical activity (PA) behaviour (i.e., engagement of planned and unplanned physical activities) among older adults. This is an important issue, because PA behaviour in the elderly is relatively low [1] when compared to what the guidelines for older adults’ PA levels proposed by the American College of Sports Medicine (ACSM) recommends [2]. Lack of motivation, illness/disability, lack of leisure time or lack of financial resources have been mentioned as some of reasons for low levels of PA participation among older adults [3]. Furthermore, factors like fear of falling and physical functioning have been shown to influence PA behaviour as well as the quality of life in elderly populations [4–7]. Perceptions of the (physical) self are modifiable and as such could be targeted in interventions to increase PA behaviour and decrease fear of falling. This is also relevant to be addressed because low PA behaviour among older adults results in functional decline, restriction of social participation, gait and balance abnormalities, reduced cognitive functioning [8], and lower vitality in old age [9, 10].

**Fig. 1:**
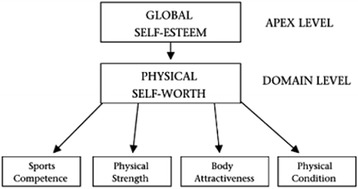
Representation of the hierarchical nature of the self in relation to physical aspects. (Source: Fox KR and Corbin CB, The physical self-perception profile: Development and preliminary validation. Journal of sport and Exercise Psychology. 1989. 11(4):408–430)

The self is multidimensional and hierarchical in nature [11]. The hierarchical nature suggests that self-esteem is at the apex. At the middle of the hierarchy are perceptions about the self in more general domains (e.g., physical, social, academic) and at the base of the hierarchy are the perceptions of behaviour and functioning in specific situations (e.g., health, strength). Figure 1 represents this hierarchy taking into consideration the physical self-perceptions only. Physical self-worth has been shown to be correlated with global self-esteem through several studies [12, 13]. Global self-esteem, on its turn, is frequently taken as a powerful indicator of mental well-being. Furthermore, physical self-perceptions and the perceived importance of aspects of the physical self have consistently been related to exercise motivation [14, 15]. Moreover, evidence has shown that people, especially youth, who report high physical competencies (i.e., high physical self-perceptions) are more likely to enjoy PA and sustain interest in continuing involvement, which, in turn, enhances motivation to be physically active [16]. However, to date relatively little is known about the association between (physical) self-perceptions and PA behaviour in older adults.

As indicated, physical and global self-perceptions have been shown to be important correlates of levels of PA behaviour in children and adolescents [17]. In older adults, perceptions of aging have been shown to be associated with preventative health behaviours (including PA uptake) [18]. In addition, in a life style physical activity intervention immediate and long-term effects were found on increased self-esteem and a number of physical self-perception domains [19]. To date, however, it is equivocal whether there is an association between global and domain specific physical self-perceptions and self-reported PA behaviour in older adults. Because interventions to enhance PA behaviour in older adults have limited effects beyond the duration of the intervention [20] it would be important to explore correlates which could assist with long-term health behaviour change [19]. Therefore, this study examined the association between the 3 levels of the model for the self (global self-esteem, physical self-esteem and factors associated with the latter (e.g., strength, endurance, flexibility and body fat)) and self-reported planned and incidental PA behaviour (i.e., PA behaviour collected via questionnaires).

Similarly, the association between global and physical self-perceptions and fear of falling has not been explored to date. Whereas higher physical self-perceptions are associated with increased PA, higher levels of fear of falling have been shown to be a predictor of activity restriction and avoidance [21]. In addition, self-perceived ratings of health, a factor determining perceptions of the physical self, has been shown in two studies in older community dwelling participants in Brazil [22] and Taiwan [23] to be associated with increased levels of fear of falling. It would also be relevant to further investigate how fear of falling is related to PA behaviour because older adults might be hesitant to try new behaviours because of fear of injury [24]. More importantly, there is a need to establish the relationship between global and physical self-perceptions and fear of falling among older adults. Hence, both concepts are based on beliefs of the ability to execute competently and safely PA behaviours. By enhancing perceptions of the (physical) self it would be expected that this results in lower levels of fear of falling. As indicated, this might provide a new portal for intervention to enhance PA behaviour in older populations.

Physical functioning including objectively measured muscle strength and gait speed have been found to be correlates of PA behaviour and fear of falling in older adults [25–28]. Reduced physical functioning is also associated with reduced quality of life [25]. For example, reduced gait speed is an independent factors for falls [29] and is associated with disability, cognitive impairment, institutionalisation, and mortality among older adults [30]. The factors associated with physical self-perceptions (e.g., fitness, coordination, strength) are also assumed to be related to objectively measured physical functioning variables. Hence, studies examining the predictive validity of physical self-perceptions questionnaires have shown correlations between actual strength and self-perceived rating of strength [31]. As such it is important to examine the association between perceptions of the physical self and actual physical functioning. In addition, previous studies have failed to include objectively measured physical functioning factors in statistical models to examine how self-perceptions can predict PA behaviour and fear of falling above and beyond these factors.

An important issue is the selection of the appropriate questionnaire to assess (physical) self-perceptions in an elderly population. The literature shows a variety of instruments used to evaluate one’s global and physical self-concept and self-perceptions [31]. Acknowledged as a leading multidimensional physical self-concept instrument [32], the Physical-Self Description Questionnaire (PSDQ) was designed to measure 11 aspects of physical self-concept [31]. The PSDQ has been modified and translated to different languages and has consistently shown sound psychometrics across cultures, including Australian, Spanish and Turkish [33]. This questionnaire has demonstrated excellent psychometric properties, including internal consistency, internal validity, and predictive validity, comparable to other self-concept instruments [34]. However, this questionnaire has not been widely used among older adults and there is not much evidence of the relationships between the physical and global self-perceptions, fear of falling and objective measures of physical function.

Therefore, the aim of this study was to examine the relationships between global and physical self-perceptions and PA behaviour, fear of falling, and objective measures of physical function among community dwelling older adults. Based on the empirical literature we expected that more positive domain specific physical self-perceptions will be associated with increased self-reported PA behaviour (H1), and lower levels of fear of falling (H2) taking into consideration the functional status of individuals. We also expect that selected subdomains of the PSDQ will be associated to self-reported PA behaviour and objectively measured physical functioning supporting its predictive validity (H3).

## Methods

### Participants

Sixty-six older people living in the community aged between 60 and 90 years old volunteered to be part of this cross-sectional study. We sought community-dwelling participants from diverse settings such as local senior organizations, retirement villages, community centres, senior clubs and associations in Melbourne. Participants were also recruited via community health promotion events and advertisement in local newspapers, magazines and online social networking media. Additionally, posters about the project were placed in healthcare facilities and places with high circulation of senior citizens and mail-out advertisements to health care practitioners in Melbourne.

The data used in this study was part of a randomized controlled trial which investigated the effectiveness of an exercise intervention in reducing older adults’ falls risk. Older adults were selected to participate if they have had one or more falls in the previous 12 months or if they were concerned about having a fall.

A fall is defined as the act of inadvertently coming to rest on the ground, floor or other lower level, excluding intentional change in position to rest in furniture, wall or any other objects [28]. Volunteers were included if they were generally active and independent in the community (i.e., older adults able to engage in daily physical activity such as stair-climbing, do their own shopping or gardening, and able to participate at least three times weekly in moderate exercise such as swimming or walking) with no more than a single point stick (i.e., use of a cane but not a walker). Participants were excluded from this study if they had: 1) any uncontrolled non-musculoskeletal conditions that would make testing difficult and uncomfortable, such as chronic obstructive airways disease and congestive heart failure; 2) a pre-existing neurological or orthopaedic condition that affects lower limb strength (e.g. polio, stroke); 3) any of the following foot conditions: partial foot amputation or ulceration or foot fractures; 4) any uncontrolled musculoskeletal conditions that may affect ambulation (rheumatoid arthritis, gout, etc.). Participants with heart problems (e.g. chest pain (angina), heart murmur, heart rhythm disturbance, heart valve disease or heart failure) were required to obtain a medical clearance from their general practitioner in order to participate in this study. Participants with any documented medical condition or physical impairment that was judged by the medical practitioner to contraindicate their inclusion were excluded.

### Protocol

All participants were fully informed about the nature of the study and signed a consent form. All testings, including assessment of strength and physical function and completion of set of questionnaires (fear of falling, physical self-perceptions and PA levels), were performed on the same day and took approximately 2 h to be completed. This study was approved by the Human Research Ethics Committee of Victoria University, Melbourne (Application ID. HRE13-215).

### Analysed measures

#### Questionnaires

Physical self-perceptions were measured using the Physical Self-Description Questionnaire (PSDQ) – Short Form [34]. The PDSQ is a 40-item questionnaire scored from 1 (false) to 6 (true) and consists of 11 subdomains: Global self-esteem, Physical self-esteem, Health, Coordination, Activity, Body fat, Sport, Appearance, Strength, Flexibility, and Endurance. Each of these subdomains can reach a maximum value of 6. The PDSQ has been shown to have good test-retest stability over a 3 month period (*r* = .81 to .94) strong factorial structure and discriminant and convergent validity [34].

The Incidental And Planned Exercise Questionnaire *(IPEQ)* for older people was used to assess PA behaviour of the participants [35]. The IPEQ is a self-report questionnaire that covers the frequency and duration of several levels of planned and incidental PA in older people. Planned activities (6-items) include planned exercise or walks whereas incidental physical activities (6-items) include day-to-day activities like housework or gardening. Total hours per week spent in both incidental and planned PA are obtained by multiplying frequency scores and duration scores. Summation of the incidental and planned PA hours per week also provided a total activity score. The IPEQ has been shown to have good test-retest reliability and concurrent and face validity [35].

The falls efficacy scale (Short FES-I) questionnaire was used to record fear of falling [36]. The FES-I consists of 7 items using a Likert scale that assesses the participant’s level of concern regarding the possibility of falling when performing certain daily activities. Items are scored from 1 = *‘not concerned at all’* to 4 = *‘very concerned’*. The total score ranges from 7 (not concerned) to 28 (severe concern) where higher scores being associated with a greater fear of falling [36]. The test–retest reliability of the Short FES-I is good (*r* = .92) [36].

#### Objective measures of strength and physical function

Hand grip strength test [37] was used to measure physical strength. Hand-grip strength is a simple, reliable, inexpensive surrogate of overall muscle strength and a valid predictor of physical disability and mobility limitation [38]. Using a TTM® digital hand dynamometer (Mentone Educational Centre, Melbourne, VIC), participants were asked to perform two maximum force trials with each hand and the best score of two attempts was recorded. Participant performed the test seated on a 43 cm high chair, feet flat on the floor, with shoulder adducted and neutrally rotated, elbow flexed at 90° and forearm in neutral and the wrist between 0 and 30 degrees extension and between 0 degrees and 15 degrees ulnar deviation [39]. The maximum values of the left- and right-hand grip measurements were summed and be used for the analysis to remove consideration of hand dominance [37].

Lower limb strength was assessed via the sit-to-stand test [40] and measurement of the strength of the knee extensor muscles using a purposely built force transducer [41]. The sit to stand test is a simple test used to measure mobility and lower limb strength [40] and is also included in fall risk assessments [42, 43]. Participants were asked to sit and stand from a 43 cm high chair as many times as possible for a period of 30 s without any assistance of the assessor. Participants were asked not to use their arms to help them rising from the chair or sitting. Thus, during the test, arms were kept crossed at the wrists and held against the chest or to the side of their body. At the signal “ready and go,” participants rose to a full stand (body straight) and then returned back to the initial seated position (fully seated with back against the chair). The score was the total number of stands executed correctly within 30 s and a full stand was counted when the participants was more than halfway up at the end of the time. Incorrectly executed stands were not counted.

The strength of the knee extensor muscles of both limbs was measured with a purposely built force transducer which was attached to the participant’s leg using a webbing strap with a Velcro fastener. The participant sat on a tall chair with a strap around the lower leg 10 cm above the ankle joint, and the hip and knee joint angles were positioned at 90 degrees. The distance from the knee joint to the strap around the ankle was measured with a tape measure. This measure was used for the calculation of torque (i.e. force [N] distance [m]). The maximum voluntary contraction was assessed during an isometric knee extension. Participants were asked to perform three maximum voluntary contractions trials on their dominant leg. The contractions last up to 5 s each, with a rest period of 1 min between each trial. The force data were stored on a portable computer. The best performance of the three trials was considered as the maximum torque and used for analysis.

Assessment of gait speed was performed with the use of the GaitRite® system (CIR System, Inc., Harverton PA) instrumented walkway system (active length of the mat: 8.75 m). Participants were asked to start from a point 3 m in front of the mat and stopped on a point 3 m behind the mat. Approximately 10 strides per participant were required to achieve reliable mean estimates of spatio-temporal gait parameters including velocity, stride and step length, and step and single support time [44]. Therefore, seven walks were recorded to allow sufficient data to be collected. Multiple practice trials were given until participants felt comfortable and could walk with consistent velocity. This was followed by seven testing trials which allowed sufficient number of strides to be recorded. Participants who used a gait aid for indoors walking were allowed to use it during the tests. Participants were wearing flat shoes during the test.

#### Data management and statistical analysis

All analyses were completed using SPSS version 22.0 and a *p* value equal or less than 0.05 was considered statistically significant. Backwards multiple regression analyses using the entry method were performed to evaluate the relationship between (1) physical self-perceptions and objectively measured physical functioning (independent variables) and self-reported PA behaviour (dependent variable); (2) physical self-perceptions and objectively measured physical functioning (independent variables) and fear of falling (dependent variable). Variables were excluded if the change in explained variance was non-significant (*p* > .05). This method would allow for the most parsimonious relationship between the independent variables and dependent variables (PA behaviour and Fear of Falling).

Age, gender and history of falls have been shown to influence physiological functioning. In particular, physiological functioning declines with increasing age [45] and is moderated by gender with men declining twice as fast compared to women [27]. These changes are also accelerated if there is a history of falls [46]. As such we first explored, using regression analysis, whether the dependent variables (PA behaviour and Fear of Falling) were influenced by these demographic data. If this was the case, they would be incorporated as covariates.

Finally, we calculated Pearson product moment correlations between factors of the PSDQ and objective measures of physical functioning and fear of falling (H3) to examine its predictive validity.

## Results

### Participants’ characteristics

Participants’ characteristics including medications, history of previous falls, levels of PA, fear of falling and physical performance characteristics are shown on Table 1. Table 2 provides an overview of the Pearson product moment correlations between the study variables.

**Table 1:** Sample means and deviations for study variables

Characteristic	Number, % or Mean ± SD	Range
*Participants (number)*	66	–
Age (Years)	71.9 ± 6.67	61–89
Number of Females in the sample	71.21	
BMI (kg/m^2^)	28.82 ± 5.19	21.4–43.6
Average Number of Medications	3.20 ± 2.15	1–10
Falls History (%, ≥ 1 fall in the last 12 months)	68.2	
Questionnaires		
*Fear of Falling*	10.97 ± 4.02	7–25
*IPEQ*		
Incidental Physical Activity	13.30 ± 5.78	1.75–26.2
Planned Physical Activity	4.14 ± 3.98	0–17.0
Total Physical Activity	17.35 ± 8.04	1.75–35.7
*PSDQ*		
Physical Activity	2.71 ± 1.56	1.0–6.0
Appearance	3.35 ± 1.39	1.0–6.0
Body Fat	3.10 ± 1.77	1.0–6.0
Coordination	4.05 ± 1.17	1.0–6.0
Endurance	2.47 ± 1.21	1.0–5.3
Global Self-Esteem	4.60 ± 0.91	1.4–6.0
Flexibility	3.61 ± 1.33	1.0–6.0
Physical Self-Esteem	3.96 ± 1.31	1.0–6.0
Health	4.98 ± 1.11	2.0–6.0
Sport	2.48 ± 1.40	1.0–6.0
Strength	3.56 ± 1.13	1.3–5.6
Strength Measures		
*Hand Grip Strength (R + L hand, Kg)*	23.79 ± 9.86	0^a^-47
*Knee Extensor Muscle Strength (Dominant Leg, N/m)*	80.32 ± 32.14	17–186
Physical Function		
*Sit to Stand (repetitions)*	10.67	0^a^-19
*Gait Speed (cm/s)*	133.19	89.5–184.9

^a^: Participant could not do the test due to knee pain and arthritis on the hand, so the value of the test for this participant was excluded from the mean. *SD* Standard deviation, *BM* Body Mass Index, *IPEQ* Incidental and Planned Exercise Questionnaire, *PSDQ* Physical Self-Description Questionnaire

**Table 2:** Prediction properties of the PSDQ subdomains with objectively measured variables of muscle strength and physical. Values are Pearson product moment correlations

	Gait speed	Grip strength	Sit-to-stand	Knee strength
Physical activity	.14	−.13	.10	−.02
Appearance	.22	.15	−.07	.03
Body fat	.08	.11	.03	.02
Coordination	.09	.19	.05	.20
Endurance/fitness	.04	.11	.24*	.15
Flexibility	.18	.12	.25*	.22
Sport competence	−.03	.21	.12	.29*
Strength	.13	.35**	.22	.42**
Health	.04	.10	.25*	.06
Physical Self-Esteem	.02	.06	.06	.12
Global Self-Esteem	−.06	.09	−.01	−.03

*: *p* < 0.05. **: *p* < 0.01. *PSDQ* Physical Self-Description Questionnaire

### Preliminary analysis

For all analyses the histograms and P-P plots indicated that the data and residuals were normally distributed. In addition, there were no outliers or collinearity (all Tolerance > .01 and VIF < 10) and the scatterplots indicated homoscedasticity. Regression analysis for Planned (*p* = .94), Incidental (*p* = .87), and Total (*p* = .86) PA did not show an association with age, gender or history of falls. However, for Fear of Falling (*p* = .02; *R* ^2^ = .15) history of falls was found to be a significant associated (Beta = .254; *p* = .04).

### H1: Predictors of PA

The backwards multiple regression analysis for Planned PA showed that the best model explained 49.3% of the variance (F(4,64) = 16.59; *p* < .001). The PSDQ subdomains Activity (Beta = .581; *p* < .001) and Coordination (Beta = −.317; *p* = .004), and sit-to-stand (Beta = .176; *p* = .05) reached significance whereas the PSDQ subdomain General Physical approached significance (Beta = .240; *p* = .06).

The best model for Incidental PA explained 15% of the variance (F(3,60) = 4.66; *p* = .005). The PSDQ subdomains Coordination (Beta = −.491; *p* = .002) and Endurance (Beta = .399; *p* = 003) reached significance whereas Global Self-Esteem approached significance (Beta = .260; *p* = .06).

Finally, the best model for Total PA explained 42.6% of the variance (F(5,59) = 8.75; *p* < .001). The PSDQ subdomains Activity (Beta = .280; *p* = .04), Coordination (Beta = −.638; *p* < .001), Endurance (Beta = .334; *p* = .01) and Flexibility (Beta = .287; *p* = .05) reached significance whereas Global Self-Esteem approached significance (Beta = .225; *p* = .06).

### H2: Predictors of fear of falling

The best backwards multiple regression model for fear of falling, controlling for falls history, was significant (F(7,57) = 8.22; *p* < .001) explaining 50.2% of the variance. The PSDQ subdomains Global Self-Esteem (Beta = −.409; *p* < .001), General Physical Self-Esteem (Beta = −.350; *p* = .009), Flexibility (Beta = −.560; *p* < .001) and Strength (Beta = .296; *p* = .03), and the objectively measured measure Knee Strength (Beta = −.356; *p* = .001) were significantly associated with fear of falling.

### H3: Association PSDQ subdomains and objectively measured physical functioning

Table 2 provides the Pearson product moment correlations between the subdomains of the PSDQ and the objectively measured physical functioning variables. There were no significant associations between the PSDQ subdomains and gait speed. Grip strength was associated with the strength factor, sit-to-stand with flexibility, sport competence and health whereas knee strength was associated with strength and health.

## Discussion

This study examined the relationship between global and physical self-perceptions, self-reported PA and fear of falling taking into consideration objective measures of physical functioning, in a sample of community dwelling older adults. Findings showed that, higher physical self-perceptions of activity and better sit-to-stand performance but lower ratings of one’s coordination was associated with higher self-reported planned PA behaviour whereas higher physical self-perceptions of endurance and global self-esteem and lower levels of coordination was associated with increased self-reported incidental PA behaviour. Similarly, increased total PA behaviour was associated with higher ratings of the physical self-perceptions of activity, endurance, flexibility and global self-esteem but lower levels of coordination (H1). More falls, lower levels of global self-esteem, domain specific physical self-esteem as well as flexibility and objectively measured knee strength was associated with increased fear of falling whereas strength had an inverse association (H2). There were also associations between some of the objectively measured physical functioning variables and the individual’s self-perceptions of the physical self, providing some predictive validity for the PDSQ (H3).

Despite research trying to identify correlates of PA behaviour, few studies have examined the influence of global or domain specific physical self-perceptions. In addition, findings on the relationship between self-perceptions and PA behaviour have been equivocal. Moore and colleagues recently showed that higher perceptions of health was strongly associated with increased PA [47] whereas other authors showed significant direct effects between perceptions of strength-, condition- and body-esteem and self-reported PA [48]. However, the latter study only measured four domains of the physical self.

The PSDQ subdomain coordination was associated with planned, incidental and total PA behaviour. Surprisingly, lower levels of coordination were associated with higher levels of self-reported planned, incidental and total PA. It is unclear why this is the case. However, we would speculate that those who perceive their coordination to be lower engage in physical activities which are of lower complexity (e.g., walking). However, this would require further investigation. It is not surprising that perceptions of activity (i.e., being active) was associated with planned and total PA but not incidental PA given that older adults who perceive themselves as more active may feel themselves more competent to engage in more structured and planned forms of PA. A study showed that older adults who perceive themselves as such reported also greater physical self-worth and global self-esteem which are directly associated with physical activity behaviour [48]. Similarly, our study also indicated that physical self-esteem was associated with planned but not incidental PA behaviour.

Incidental and total PA, on the other hand, was associated with perceptions of one’s endurance (i.e., not tiring when exercising hard) and global self-esteem. Higher levels of physical fitness and endurance have been shown to indirectly influence PA behaviour through increased exercise related self-efficacy [49]. Global self-esteem has been found to decline in older individuals [50]. In addition, self-esteem is associated with well-being, health, life-satisfaction and quality of adaptation [51]. Also, it is associated with social integration and ability to cope with physical and cognitive decline happening in older age [51]. Conversely, other studies have suggested that not all older adults are likely to be exposed to declines in their self-esteem with some maintaining it fairly stable or even increasing their levels throughout adulthood [52]. It is also believed that self-esteem could change for different older adults in different directions [53] and one way that it could be improved would be through interventions [19, 54]. Finally, increased levels of total PA behaviour was associated with higher perceptions of one’s flexibility.

Overall, our study showed that global and specific physical self-perceptions had closer associations with self-reported planned and incidental PA than objective measures of physical functioning. Only the sit-to-stand physical functioning test was associated with planned PA. This is an important finding and provides support for the multidimensional and hierarchical Exercise and Self-Esteem Model [55] in explaining the relationship between self-perceptions and PA. It suggests that PA influences self-efficacy (i.e., the belief in your ability to complete a physical task) – not measured in the present study – which, in turn, impacts areas of physical competence (e.g., coordination, endurance/fitness and activity) which then, directly and indirectly (through physical self-worth), influences global self-esteem [47]. Research has previously also showed that the main barriers to engaging in PA among older people are attitudinal [56]. It is therefore relevant that the non-physical aspects of PA, such as (physical) self-perceptions are also taken into account when designing intervention programmes [56]. Hence, through enhancing individuals global self-esteem and domain specific physical self-perceptions, PA levels might be increased either directly or indirectly [19].

Fear of falling tends to constrain and limit older people’s activity and mobility which in turn can reduce physical conditioning and reducing muscular strength [46]. Decreased mobility and muscle atrophy lead to more accidental falls, which in some studies has been associated with increased fear of falling [57]. We found that increased number of falls resulted in increased fear of falling of falling and controlled for this in our analysis. All 3 levels of the model of the self (global self-esteem, physical self-esteem and its factors flexibility and strength) as well as actual knee strength was associated with 50.2% of variance in fear of falling. Like coordination and PA behaviour, perceptions of strength had an inverse association and it is unclear why this is the case.

Few studies have examined the role of global self-esteem or a broad spectrum of perceptions of the physical self in relation to fear of falling. There is some support for the influence of objectively measured factors on fear of falling. One previous study showed that reduced physical functioning and slower gait speed was associated with higher fear of falling [58]. In addition, elderly individuals who have irrational fears are more likely to interpret physical impairments negatively with the potential to influence physical functioning (e.g. fear induced co-contraction) [59]. However, this present study is the first one which has demonstrated that perceptions of global self-esteem, physical self-esteem, flexibility and strength are also important in levels of fear of falling. Surprisingly, the factors associated with fear of falling have been shown to be different from those related to actual falls [60]. Falls are related to age, gait speed and being depressed whereas fear of falling has been associated with age, cognitive impairment, lower social activity and being female [60]. Our findings also indicated that a history of more falls was associated with higher levels of fear of falling.

Few studies have examined the predictive validity of the PSDQ. Similar to Brewer and Olson [61], we found a moderate correlation between the strength factor of the PDSQ and grip and knee strength. The latter was also associated with rating of health. The sit-to-stand task is generally considered a measure of functional performance and has been shown to be influenced by a number of physiological and psychological factors [62]. The present study suggests that the sit-to-stand test is associated with the flexibility, sport competence and health subdomains of the PSDQ. Although gait speed has been shown to be associated with health related outcomes in normally functioning older individuals [63] we did not find an association between gait speed and any global and domain specific physical self-perceptions. This is a surprising finding and further research is required to examine this issue. Our findings provide some support for the predictive validity of the PSDQ in a sample of community dwelling older adults.

It has been unclear whether all domains assessed in the PSDQ are relevant to older individuals. Consistent with previous research, the present study showed that older individuals scored lower for the subdomains sport [19] and endurance [34] compared to young adults. However, these latter authors also showed lower scores for health and body fat whereas some other authors found lower scores for flexibility and coordination [64]. Ratings of the self are very much influenced by the frame of reference used by individuals as well as actual performance. Thus, lower average scores in a number of areas of physical self-worth can be due to declines with age and older adults’ ratings of global self-esteem and physical self-worth are more likely to be related to social comparisons with individuals of similar age [34].

### Practical implications

Overall, we found an association between global and domain specific physical self-perceptions. This finding might provide a pathway for developing strategies for the adoption and maintenance of PA behaviour in older individuals as previously suggested [63]. This could be accomplished by matching exercise interventions to the self-perceptions of individuals or by developing interventions which influence physical self-perceptions in order to individuals becoming more likely to take-up and maintain a PA routine. For example, practitioners could consider interventions which match low physical self-perceptions to coordination or endurance. The former might result in individuals being advised in engaging in Tai Chi type of activities whereas the latter might result in advice to engage in activities like walking or cycling.

Previous research has suggested that a person’s attitudes, actions and behaviours are guided by their beliefs [65, 66]. Therefore, as also supported by our findings, interventions aimed at reducing falls or fear of falling among older adults should take into consideration global and domain specific physical self-perceptions. This could be accompanied by goal-setting [67], life coaching [68] and behaviour change strategies [69, 70] to address engagement and adherence to interventions aiming to reduce risk of falls and fear of falling [71]. For example, a recent study has demonstrated that an intervention that combines cognitive-behaviour strategies can help older adults to manage their fear of falling, falls, decrease their depressive inclination, and enhance their mobility and muscle strength [72].

### Limitations

While this study provides useful information about the relationships between perceptions global and physical self-perceptions, self-reported PA, fear of falling and objective measures of physical function among older adults several limitations are acknowledged. Firstly, we used a relatively small convenient sample. Although this makes generalisation of findings problematic, the participants included in the study were recruited from an area of Melbourne which is likely to resemble healthy older adults in Australia in general. Secondly, a disparity in gender was present where more females volunteered to be part of this study. Similarly, we had an unequal number of fallers/non fallers recruited. Therefore, further research is needed with a more homogeneous sample of older adults. Also, there is still some unexplained variance in the evaluated measures and questionnaires which indicates that other subdomains may play a role in the investigated relationships. We would like to acknowledge that in our cross-sectional study we assumed that self-perceptions predicted PA behaviour. However, we cannot establish cause and effect and it is likely that there is a reciprocal relationship between self-perceptions and PA behaviour. Finally, the information regarding the levels of physical activity of participants was evaluated and accounted for via the Incidental and Planned Exercise Questionnaire. An alternative to minimize errors due to inaccurately reported PA behaviour would be to measure physical activity behaviour objectively. Therefore, for future studies, an objective method for assessing PA behaviour such as accelerometers or pedometers would be recommended to be included.

## Conclusion

Findings showed that global and physical self-perceptions are associated with planned (49.3%) and incidental (15%) PA behaviour and fear of falling (50.2%). Some of the objectively measured physical functioning variables were associated to the individual’s self-perceptions of the physical self, providing some predictive validity for the PDSQ. The findings of this study come to corroborate that the belief system of older adults ideally need to be taken into consideration when designing interventions that aim to increase PA behaviour, reduce fear of falling or actual fall. Coupling that with goal-setting, life coaching and behaviour change strategies would also be beneficial to address engagement and adherence to such interventions.

## Abbreviations

IPEQ: Incidental and planned exercise questionnaire
PA: Physical activity
PSDQ: Physical self-description questionnaire
RCT: Randomised controlled trial
Short-FES-I: The falls efficacy scale
